# Method for the determination of parameters in the sintering process of mixtures of the elemental powders Fe-Cr and Fe-Cr-Ni

**DOI:** 10.1016/j.mex.2019.08.009

**Published:** 2019-08-23

**Authors:** Tárique Hernandez Schneider, Luciano Volcanoglo Biehl, Ederson Bitencourt das Neves, Jorge Luiz Braz Medeiros, José de Souza, Fábio Augusto Dornelles do Amaral

**Affiliations:** a Universidade Federal do Rio Grande do Sul, PPG3M, UFRGS, Porto Alegre, RS, Brazil; b Universidade Federal de Rio Grande (FURG), PPMec Rio Grande, RS, Brazil; c Universidade Federal de Pelotas, PPGCEM, Pelotas, RS, Brazil; d Fundação Liberato, Diretoria de Pesquisa e Produção Industrial (DPPI), Novo Hamburgo, RS, Brazil

**Keywords:** Method for the determination of parameters in the sintering process of mixtures of the elemental powders Fe-Cr and Fe-Cr-Ni, Sintering, Elemental metallic powders mixture, Diffusion of metallic powders, Chromium, Stainless steel

## Abstract

The achievement of varied metal alloys through the sintering of mixtures of elemental metallic powders can provide great flexibility of production of these alloys. The possibilities of controlling the composition and the resulting properties of the alloy are of great importance for metallurgy. Otherwise the alloys involving chromium offer many difficulties for their attainment through the process of powder metallurgy (PM). In the formation of alloys with chromium (Cr), an oxide layer that prevents the ideal contact between the particles is formed. Hence the need for researches that study sintering processes and that can solve this difficulty. With this objective, this paper presents a method to determine parameters and describe the sintering process of mixtures of the elemental metallic powders Fe-Cr and Fe-Cr-Ni, assessing the elements’ diffusing capacity and the possible alternative composition of an alloy. The method used Scanning Electron Microscopy (SEM) associated with Energy Dispersive Spectrometry (EDS), X-ray diffraction (XRD) and Vickers microindentation hardness analysis (HV). For the microstructural characterization, we used optical microscopy (OM) and SEM. And EDS mapping was applied for the analysis of the elements’ dispersion.

• The method makes it possible to define the ideal sintering time to obtain a good diffusion of the mixtures of the metallic powders Fe-Cr.
• With the method, it is possible to verify the dispersion of the powders and whether the microstructure generated is similar to the ones of prealloyed powders.
• With the applied procedures, the conditions for the formation of Fe-Cr alloys can be obtained by mixing dissociated and diffusion-bonded elemental metallic powders.

## Graphical abstract

**Figure:**
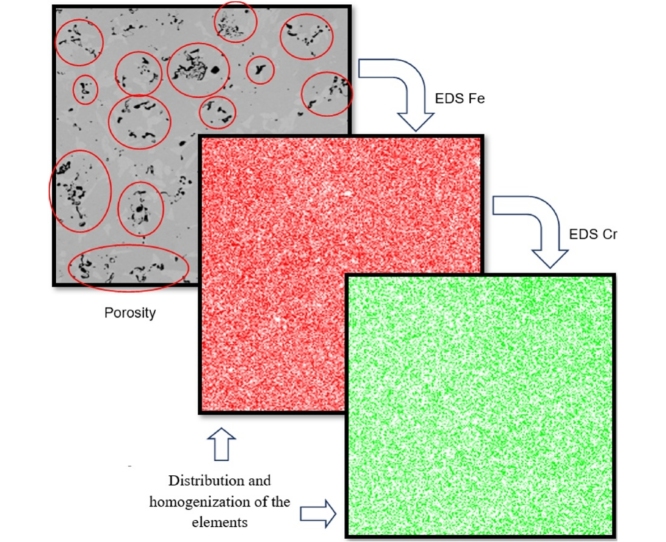

Specifications Table

**Table:** 

Subject Area:	*Materials Science*
More specific subject area:	*Powder metallurgy (PM)*
Method name:	*Method for the determination of parameters in the sintering process of mixtures of the elemental powders Fe-Cr and Fe-Cr-Ni*
Name and reference of original method:	*ASTM International, ASTM B925 Standard Practices for Production and reparation of Powder Metallurgy (PM) Test Specimens, West Conshohocken, USA, 2008.* *DAVIES, G. A., PONTER, A. B., I. A., MENZIES, I. A. The Diffusion of Chromium in Iron and low Carbon Steels, Acta Metallurgica, Vol. 15, 1967.* *KING, P., LINDSAY, B. Chromium Steels for high performance PM applications, Hoeganaes Corporation, Presented at PowderMet, 2007.*
Resource availability:	*N/A*

## Method details

### Method

**Fig. 1:**
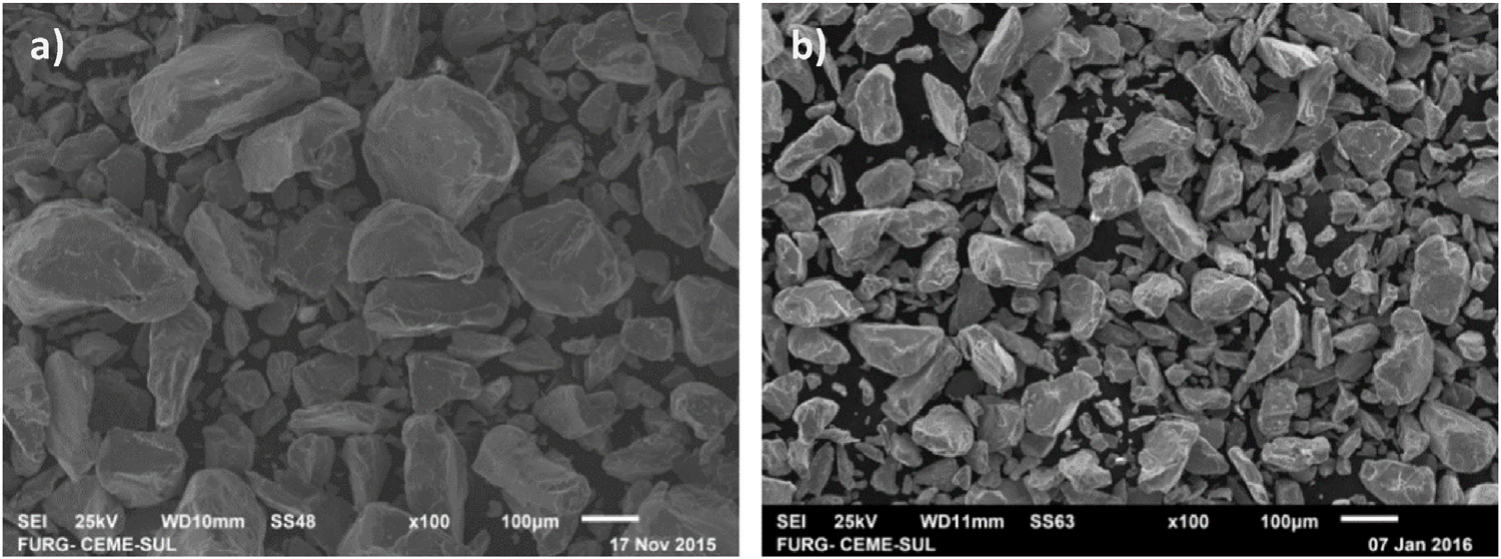
Characterization through EDS of Cr powder supplied and sieved. a) Cr elementary powder; b) Cr screened powder.

The method to determine parameters and describe the sintering process of mixtures of the elemental metallic powders Fe-Cr and Fe-Cr-Ni, evaluating the elements’ diffusing capacity, consists of the following steps:

1 – Energy Dispersive Spectrometry (EDS) analysis of the metallic powders Fe, Cr and Ni, with a Scanning Electron Microscope (SEM), to verify the morphology of the particles and their respective compositions. Then an X-ray diffraction analysis is executed for the complementary characterization of the composition. The parameters to be used are: copper anodes and wavelength λ = 015,406 nm and angular pitch of 002° [1]. The results of the diffraction patterns and the expected position of the peaks for each element in their respective crystal structure are then verified. The morphology and degree of homogeneity of the particles have an important effect on the characteristics of the compact [2]. Fig. 1 presents images of the analysis results obtained with the EDS technique, of the Cr powder supplied and after being sieved.

**Fig. 2:**
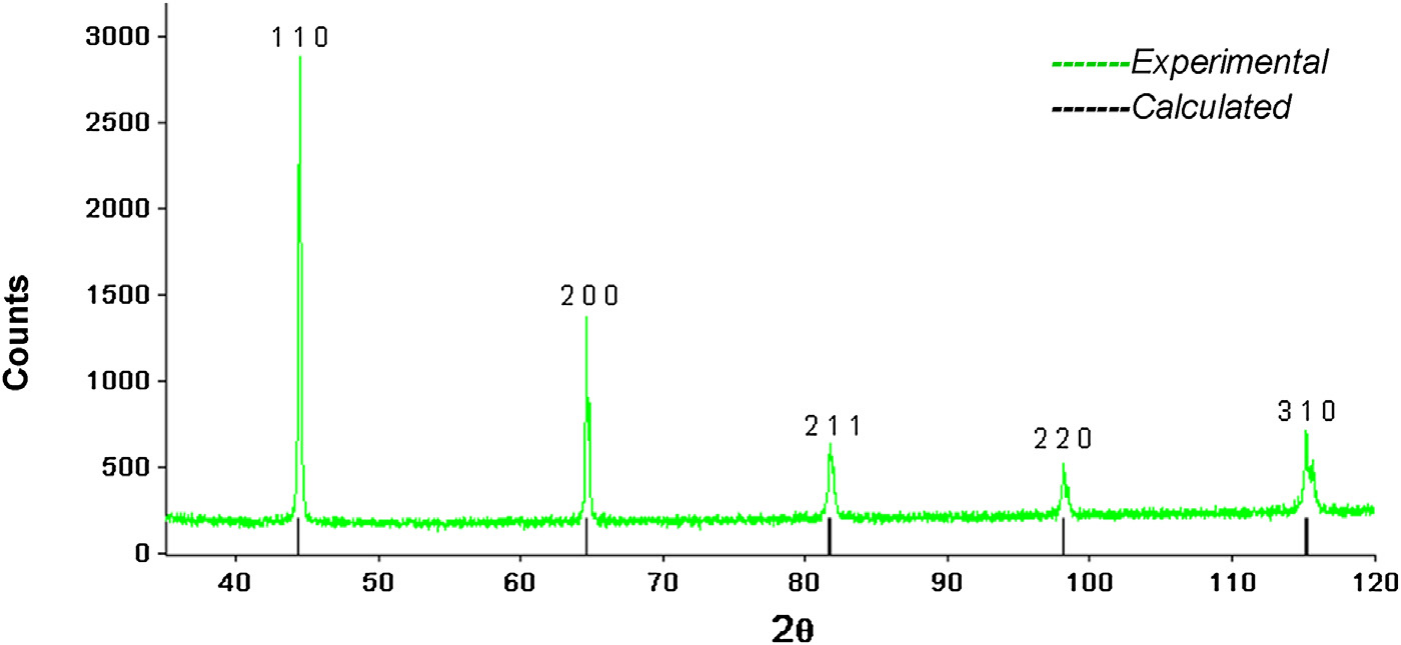
Chrome powder diffractograms.

The XRD analysis of the powders allows us to verify the peaks’ expected position. In Fig. 2, the bars in the axis of the graph represent the peaks’ expected position.

2 – Development of samples with individual weighing of each powder (Fe, Cr and Ni). The procedure consists in weighing groups of 90 g for each composition, representing a series of tests. The metal powders should then be inserted into a Y blender and mixed for 60 min at 45 RPM for the homogeneous dispersion of the components. Each mixture should then be weighed into 10 g samples to compose the samples for testing.

3 – The compression of the samples must follow the process of uniaxial application of a load on a rigid steel die, with an internal diameter of Ø = 18.75 mm [3]. A press with capacity for 30 tonnes or more, duly calibrated, should be used. This procedure follows the recommendations of the norm ASTM B925-15 section 7, which identifies the procedure for the making of PM samples, namely: (a) the lower punch, smaller, is inserted into the die, supported by the elastic spacer; (b) the powder is inserted into the cavity, then the upper punch; (c) the assembly is positioned in the press and a load of 1 t is applied to support the die and remove the spacer; (d) the load is applied to its final value at a rate of approximately 20 MPa/second, not exceeding 10 s of permanence; and (e) the pressure is withdrawn, the lower punch is removed and an upper spacer is positioned for the extraction of the compact, executed in the same equipment [1].

**Fig. 3:**
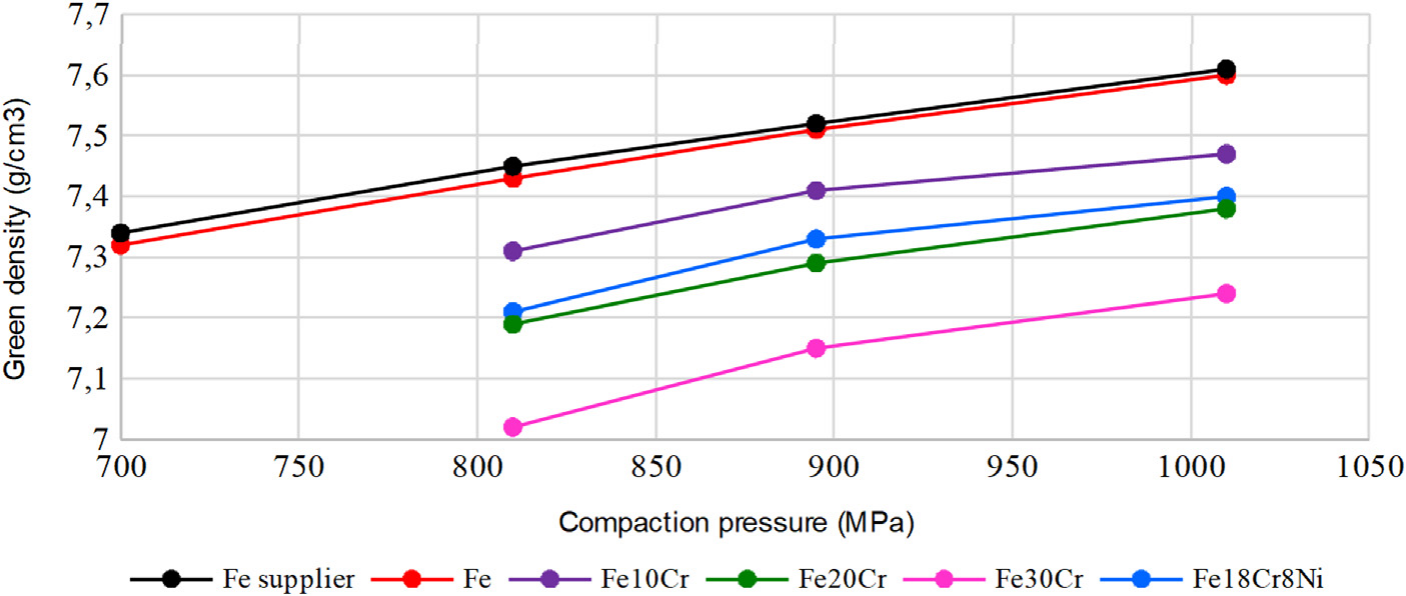
Compaction curve of powder mixtures.

4 – The compaction curve should be determined for the Fe powder as it was received by the supplier and for mixtures of 10%, 20% and 30% Cr. The process consists of the compaction of samples according to the process described, with increasing loads of 700 MPa to 1000 MPa. Each sample should have its green density determined, and then its values corresponding to the applied load should be located in a graph; that should be done for each different composition. To determine the Fe graph, the values supplied by the manufacturer should be compared to the measured values of the densities corresponding to the tensions of 700 MPa, 800 MPa, 900 MPa and 1000 MPa. For the Cr mixtures, the densities for the loads involved in the study of 800 MPa, 900 MPa and 1000 MPa should be determined. The compaction curve of the metal powders should be determined. An example is shown in Fig. 3.

The analysis of the different mixtures shows a tendency of reduction of the densities with the increase in the proportion of Chromium [4]. However, the curve continues with the same trend. The curve of the mixture with Nickel was located just above the mixture with 20% Chromium. This was expected, given that nickel particles are smaller than those of the other elements and can attach themselves to the pores of the material, making it more dense.

**Fig. 4:**
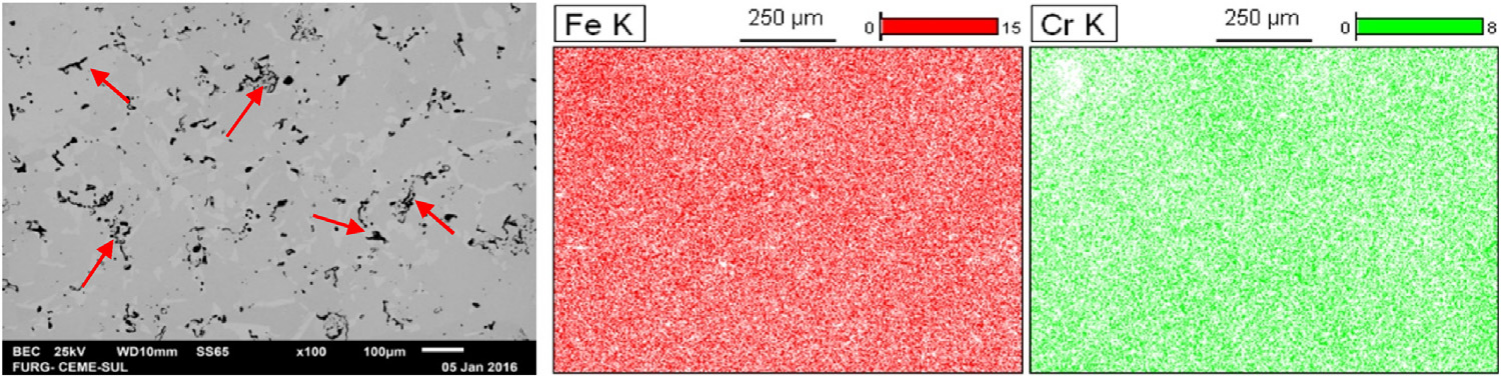
80%Fe 20%Cr 800 MPa.

The SEM analyses and EDS mapping of the sintered samples allow us to analyze the porosity of the material, which can be noticed in the dark spots. Fig. 4 shows the presence of more dark regions in comparison with the samples that had more immersion time. That is due to the fact that longer immersion times allow for a greater degree of sintering, so that the empty spaces coalesce and close up, as mentioned.

**Fig. 5:**
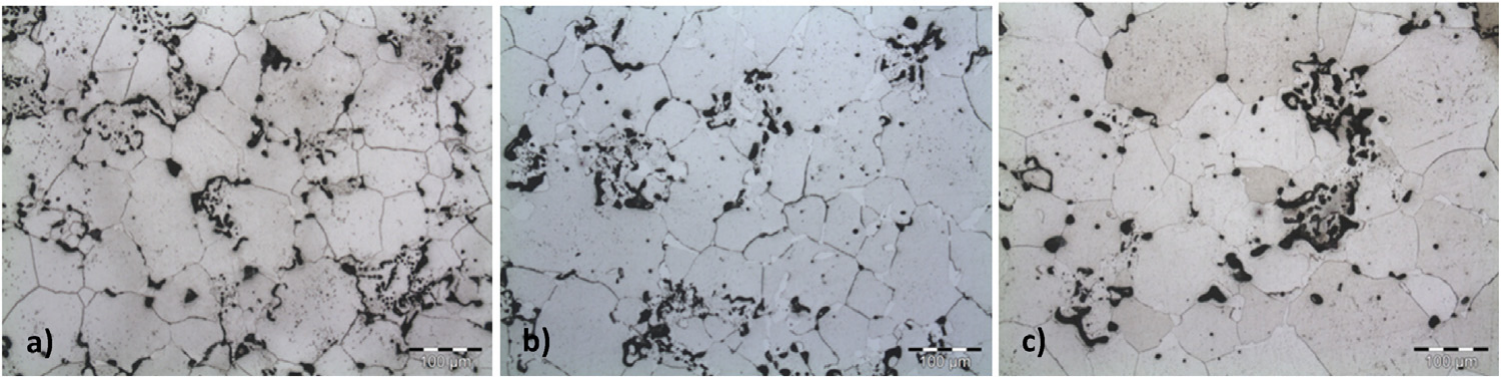
Optical microscopy of the sample 80%Fe 20%Cr 800 MPa for a) 60, b)180 and c) 360 min.

5 – After the compaction process, the samples should be identified and conducted to the sintering process. The sintering of the components needs to occur in a furnace with heating capacity of 1200 °C under argon atmosphere. The furnace should be programmed to heat up to the sintering temperature when it is already inert with argon. When the temperature is reached, the parts must be inserted into the furnace. To avoid oxidation at high temperatures, the samples should be placed on an ASTM 304 steel plate with similar chromium content, to avoid high temperature corrosion or the segregation of components [2]. The gas should be leaked at 4 l/min. The heating rate needs to be measured by positioning an auxiliary thermocouple right above the samples, controlled by a thermometer with a minimum resolution of 0,1 °C and referenced at 1200 °C, according to the calibration certificate. The time taken for the samples to reach the final temperature must be observed, and then the samples should be kept in the furnace for 60, 180 and 360 min. After the specified time has been taken, the parts should be removed from the furnace, still in the sintering temperature, and left to cool down in the external environment, in the air. Fig. 5 shows the optical microscopy quality of the specimen with 80% Fe and 20% Cr compacted with 800 MPa and sintered in Argon atmosphere (Ar) for 60, 180 and 360 min.

**Fig. 6:**
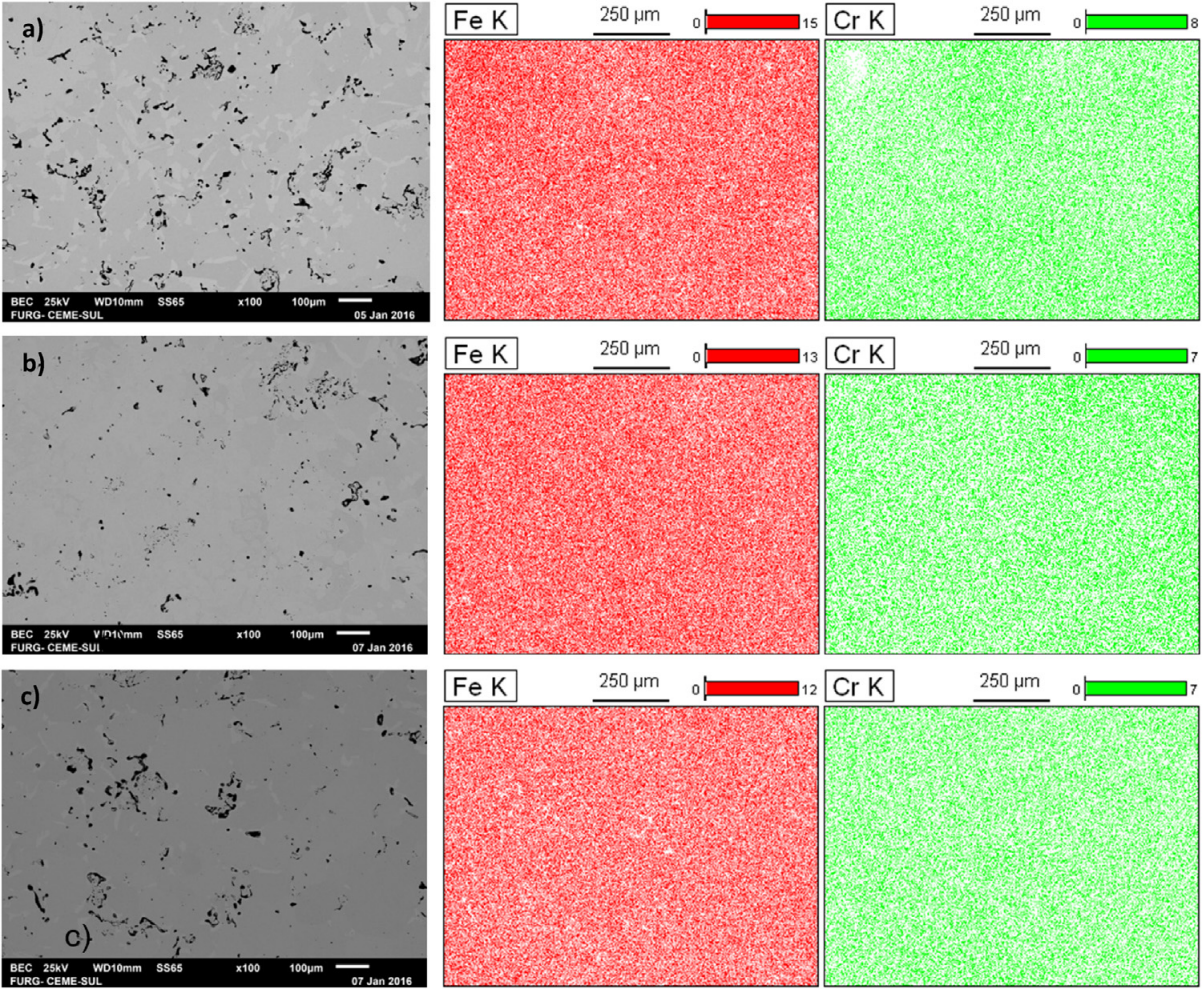
SEM images with 80% Fe and 20% Cr compacted with 800 MPa. a) 60, b) 180, and c) 360 min.

6 – SEM analyses and EDS mappings of the sintered samples should be executed. The results of the scanning electron microscopy and the mapping of the region via EDS will be obtained through the variation of retro-scattered electrons, followed by the mapping of the elements of interest in the same region photographed. All samples should be analyzed with composition variation, compaction variation and sintered at 1200 °C for 60, 180 and 360 min. Fig. 6 shows SEM images with 80% Fe and 20% Cr compacted with 800 MPa and sintered in Argon atmosphere (Ar) for a) 60, b) 180, and c) 360 min.

In Table 1, below, shows the average values of Vickers hardness and the standard deviations associated with each sample.

**Table 1:** Hardness values for the samples.

Mixture	Stress	Time (min)	Hardness (HV)	Deviation
Fe 20Cr	800 MPa	180	176.67	11.85
		360	150.72	19.55
	1000 MPa	180	160.04	9.64
		360	197.06	16.39

Fe 30Cr	800 MPa	180	214.34	18.97
		360	212.86	14.22
	1000 MPa	180	190.2	12.08
		360	194.34	16.92

The results analysis demonstrated a homogeneous dispersion with a narrow range in hardness values in all samples. It is also possible to observe that there was minimal deviation of the hardness values within the same sample. This result suggests that even in samples of shorter compaction time and tension, there was a sufficient chromium distribution to generate no significant changes in the hardness profile [5].
